
JOURNAL INFORMATION
==============================
NLM Title Abbreviation: ScientificWorldJournal
Journal ID: ScientificWorldJournal
NLM Journal ID: 101131163
Journal ID: TSWJ

The Scientific World Journal

ISSN: 2356-6140
EISSN: 1537-744X
Publisher: Wiley

ARTICLE INFORMATION
==============================
PMCID: PMC5900481
PMID: 29861694
DOI: 10.1100/tsw.2007.105
Article ID: 676382
Article version: 1
Subjects: Table of Contents

IUFRO Research Group 7.01 “Impacts of Air Pollution and Climate Change on Forest Ecosystems” Riverside, California, September 10-15, 2006

Publication date: 2007
Electronic publication date: 2007 Mar 2
Volume: 7
First page: 264
Last page: 266
Copyright: Copyright © 2007 TheScientificWorldJOURNAL
Copyright year: 2007
License: This is an open access article distributed under the Creative Commons Attribution License, which permits unrestricted use, distribution, and reproduction in any medium, provided the original work is properly cited.
License URL: https://creativecommons.org/licenses/by/3.0/

==============================
Proceedings: Impacts of Air Pollution and Climate Change on Forest Ecosystems
*TheScientificWorldJOURNAL* (2007) 7(S1), 264–266
ISSN 1537-744X; DOI 10.1100/tsw.2007.105

TheScientificWorldJOURNAL
www.thescientificworld.com

### IUFRO Research Group 7.01 "Impacts of Air Pollution and Climate Change on Forest Ecosystems"
### Riverside, California, September 10-15, 2006

### Forests under Influence of Air Pollution, Climate Change and Urban Development

### EDITORS

***Andrzej Bytnerowicz, Michael Arbaugh, Mark Fenn, Robert Heath, Elena Paoletti, and Benjamin Sanchez Gimeno***

### Contents

Impacts of Air Pollution and Climate Change on Forest Ecosystems — Emerging Research Needs 1-8
*Elena Paoletti, Andrzej Bytnerowicz, Chris Andersen, Algirdas Augustaitis, Marco Ferretti, Nancy Grulke, Madeleine S. Günthardt-Goerg, John Innes, Dale Johnson, Dave Karnosky, Jesada Luangjame, Rainer Matyssek, Steven McNulty, Gerhard Müller-Starck, Robert Musselman, and Kevin Percy*

**Diagnosis, Monitoring and Evaluation**

Secondary Air Pollutants and Forests — New Perspectives 9-14
*J. Neil Cape*

The Use of Critical Levels for Determining Plant Response to Ozone in Europe and in North America 15-21
*Robert C. Musselman and Allen S. Lefohn*

Forest Health Status in Europe 22-27
*Martin Lorenz and Volker Mues*

Forest Health Status in North America 28-36
*Borys Tkacz, Ben Moody, Jaime Villa Castillo*

Trend in Ambient Ozone and an Attempt to Detect Its Effect on Biota in Forest Ecosystem. Step I of Lithuanian Studies 37-46
*Algirdas Augustaitis, Ingrida Augustaitiene, Almantas Kliucius, Gintautas Mozgeris, Gintaras Pivoras, Rasele Girgzdiene, Kestutis Arbaciauskas, Irena Eitminaviciute, and Reda Mazeikyte*

Contribution of Ambient Ozone to Changes in Scots Pine Defoliation. Step II of Lithuanian Studies 47-57
*Algirdas Augustaitis, Ingrida Augustaitiene, Almantas Kliucius, Rasele Girgzdiene, and Dalia Sopauskiene*

Did the Ambient Ozone Affect Stem Increment of Scots Pines (*Pinus sylvestris* L.) on Territories under Regional Pollution Load? Step III of Lithuanian Studies 58-66
*Algirdas Augustaitis, Ingrida Augustaitiene, Gintautas Cinga, Juozapas Mazeika,*

©2007 with author.
Published by TheScientificWorld; www.thescientificworld.com

*Romualdas Deltuvas, Romualdas Juknys, and Adomas Vitas*

Ozone in Spain's National Parks and Protected Forests 67-77
*María J. Sanz, Francisco Sanz, Vicent Calatayud, and Gerardo Sanchez-Peña*

Monitoring of Ozone Risk for Forest Stands in the Czech Republic – Results of from 2005 Survey 78-83
*Vít Šrámek, Radek Novotný, Emilie Bednářová, and Hana Uhlířová*

Analysis of Climate Change Affecting German Forests by Combination of Meteorological and Phenological Data within a GIS Environment 84-89
*Winfried Schröder, Roland Pesch, Gunther Schmidt, and Cordula Englert*

Visible Foliar Injury and Physiological Responses to Ozone in Italian Provenances of *Fraxinus excelsior* and *F. ornus* 90-97
*Nicla Contran and Elena Paoletti*

Air Pollution Distribution Patterns in the San Bernardino Mountains of Southern California: a 40-Year Perspective 98-109
*Andrzej Bytnerowicz, Michael Arbaugh, Susan Schilling, Witold Fraczek, Diane Alexander, and Philip Dawson*

**Mechanisms of Action**

Alterations of the Biochemical Pathways of Plants by the Air Pollutant Ozone: Which are the True Gauges of Injury? 110-118
*Robert L. Heath*

Parameterization of the Stomatal Component of the $DO_3SE$ Model for Mediterranean Evergreen Broadleaf Species 119-127
*Rocío Alonso, Susana Elvira, María J. Sanz, Lisa Emberson, and Benjamín S. Gimeno*

Ethylenediurea (EDU) Affects the Growth of Ozone-Sensitive and Tolerant Ash (*Fraxinus excelsior*) Trees under Ambient $O_3$ Conditions 128-133
*Elena Paoletti, Nicla Contran, William J. Manning, and Francesco Tagliaferro*

Chronic *vs.* Short-Term Acute $O_3$ Exposure Effects on Nocturnal Transpiration in Two Californian Oaks 134-140
*N.E. Grulke, E. Paoletti, and R.L. Heath*

Changes in the Essential Oil Composition in the Needles of Scots Pine (*Pinus sylvestris* L.) Under Anthropogenic Stress 141-150
*Asta Judzentiene, Aida Stikliene, and Eugenija Kupcinskiene*

**Atmospheric Deposition, Soils and Nutrient Cycles**

Nitrogen Accumulation in Forests. Exposure Monitoring by Mosses 151-158
*Roland Pesch, Winfried Schröder, and Gunther Schmidt*

Modeling of Nitrogen Dynamics in an Austrian Alpine Forest Ecosystem on Calcareous Soils: A Scenario-Based Risk Assessment under Changing Environmental Conditions 159-165
*Friedl Herman, Stefan Smidt, Klaus Butterbach-Bahl, Michael Englisch, Ernst Gebetsroither, Robert Jandl, Klaus Katzensteiner, Manfred Lexer, Friederike Strebl, and Sophie Zechmeister-Boltenstern*

Soil $CO_2$ Efflux in a Mixed Pine-Oak Forest in Valsaín (Central Spain) 166-174
*Rosa Inclán, Daniel De la Torre, Marta Benito, and Agustín Rubio*

Smog Nitrogen and the Rapid Acidification of Forest Soil, San Bernardino Mountains, Southern California 175-180
*Yvonne A. Wood, Mark Fenn, Thomas Meixner, Peter J. Shouse, Joan Breiner, Edith Allen, and Laosheng Wu*

Chemometric study of bulk precipitation and throughfall in different age of spruce stands on Potok Dupniański catchment within 1999-2003 (Silesian Beskid-Southern Poland) 181-191
*Stanisław Małek and Aleksander Astel*

Atmospheric Heavy Metal Input to Forest Soils in Rural Areas of Denmark 192-197
*M.F. Hovmand and Kaare Kemp*

Calculation of Theoretical and Empirical Nutrient N Critical Loads in the Mixed Conifer Ecosystems of Southern California 198-205
*Joan Breiner, Benjamin S. Gimeno, and Mark Fenn*

Air Pollution and Watershed Research in the Central Sierra Nevada of California: Nitrogen and Ozone 206-221
*Carolyn Hunsaker, Andrzej Bytnerowicz, Jessica Auman, Ricardo Cisneros*

**Genetics, Adaptation and Succession**

Pre-Exposure to Ozone Predisposes Oak Leaves to Attacks by *Diplodia corticola* and *Biscogniauxia mediterranea* 222-230
*Elena Paoletti, Naldo Anselmi, and Antonio Franceschini*

Linking Increasing Drought Stress to Scots Pine Mortality and Bark Beetle Infestations 231-239
*Matthias Dobbertin, Beat Wermelinger, Christof Bigler, Matthias Bürgi, Mathias Carron, Beat Forster, Urs Gimmi, and Andreas Rigling*

Impacts of Elevated Atmospheric $CO_2$ and $O_3$ on Paper Birch (*Betula papyrifera*): Reproductive Fitness 240-246
*Joseph N.T. Darbah, Mark E. Kubiske, Neil Nelson, Elina Oksanen, Elina Vaapavuori, and David F. Karnosky*

Patterns of Understory Diversity in Mixed Coniferous Forests of Southern California Impacted by Air Pollution 247-263
*Edith B. Allen, Patrick J. Temple, Andrzej Bytnerowicz, Michael J. Arbaugh, Abby G. Sirulnik, and Leela E. Rao*
